# Clinical and Demographic Predictors of Survival in Elderly Burn Patients Aged 65 and Older: A Retrospective Analysis in an Appalachian Cohort

**DOI:** 10.7759/cureus.82283

**Published:** 2025-04-15

**Authors:** Armein Rahimpour, Lauren Clower, Ashton McDonald, Sharday Young, Heather Reeves, Thomas Cassier, Curtis W Harrison, Paul Bown, Rahman Barry

**Affiliations:** 1 General Surgery, Marshall University Joan C. Edwards School of Medicine, Huntington, USA; 2 Medicine, Marshall University Joan C. Edwards School of Medicine, Huntington, USA; 3 Plastic and Reconstructive Surgery, Marshall University Joan C. Edwards School of Medicine, Huntington, USA

**Keywords:** appalachia, burn mortality, elderly burn patients, inhalation injury, survival outcomes, total burn surface area (tbs)

## Abstract

Background

Elderly burn patients, particularly those aged 65 and older, represent a vulnerable population with unique clinical challenges. This study aims to evaluate clinical and demographic factors associated with survival outcomes in elderly burn patients within an Appalachian cohort.

Methods

A retrospective analysis was conducted on 198 patients aged ≥65 years admitted to a tertiary care center in Appalachia. Patient demographics, comorbidities, burn characteristics, and outcomes were analyzed. Bivariate analyses, including chi-square tests, t-tests, and Mann-Whitney U tests, were used to identify factors associated with discharge status (alive vs. deceased).

Results

The mean age of the cohort was 72.6 ± 6.1 years, with 65.2% male participants. The overall survival rate was 85.9%. Key findings included a statistically significant association between age and survival (p = 0.031), with older patients more likely to experience mortality. Inhalation injuries were strongly associated with increased mortality (p < 0.001). Patients who died had a higher median total burn surface area (TBSA) of 30.0% (interquartile range (IQR): 10.0-50.0) compared to 5.0% (IQR: 1.0-10.0) in survivors (p < 0.001). Other variables, including comorbidities, BMI, and burn source, did not demonstrate statistically significant associations with mortality.

Conclusions

Age, inhalation injury, and TBS were significant predictors of mortality in elderly burn patients. These findings underscore the importance of targeted interventions and resource allocation for high-risk patients, particularly in underserved regions like Appalachia. Further research is needed to improve outcomes in this vulnerable population.

## Introduction

Burn injuries are the fourth most common type of traumatic injury globally and are associated with high rates of morbidity and mortality, with close to 200,000 dying annually [1-2]. These injuries pose issues in the healthcare system due to significant costs, the need for specialized training for the staff, specialized medical equipment, and long periods of rehabilitation [3-4]. Estimated healthcare costs per burn patient in high-income countries was $88,212 between 1950 and 2012 [4]. Total burn surface area (TBSA) was the main predictor for higher costs for burn patients with a price of $4,097 per 1% of TBSA burned [4]. The goal of care for these patients is to decrease the length of hospital stay as an increased length of hospital stay is associated with lower quality of care, higher costs, and decreased rates of reimbursement from insurance companies [3]. 

Burns in elderly patients are more common in developed countries such as the U.S [5]. In 2016, these cases accounted for approximately 20% of burn injuries [5]. Older adults are less likely to be counseled on burn prevention by their primary care providers and are more difficult to treat due to comorbidities upon presentation [6]. They are more prone to burn injuries due to thinner skin, decreased skin sensation, impaired coordination and balance, and impaired reaction time [6-7]. Thinning of the dermis results in deeper burns, and these patients can contract organisms with increased virulence [8-9]. Deeper burns can present with more complications and may require surgical intervention for healing if 20% or more of TBSA is affected [1-2]. Infections, fluid loss, shock, and sepsis are all concerns in this patient demographic [1-2]. It is important, moving forward, to prevent these injuries by improving medical care and implementing refined prevention strategies as patients in this age group have increased rates of mortality [6,10].  

West Virginia, in particular, has an increasing elderly population, and the majority of the state is classified as rural [11]. This presents unique challenges for patients with limited access to nearby medical facilities and longer traveling distances. WV households have a higher percentage of families living in poverty or just above the poverty line compared to other states [12]. Rural areas compared to urban areas have lower median incomes, decreased job availability, increased rates of poverty, inadequate transportation, more risky health behaviors, and higher rates of food insecurity [13]. These disparities are more prominent in rural areas and need to be addressed for more impartial healthcare [14]. 

For patients with severe burn injuries, there are limited resources available to them in WV. Cabell Huntington Hospital in Huntington, WV, has the only burn intensive care unit (BICU) in the state with a limited capacity of six beds in the unit. This study analyzed data collected over a six-year period for elderly patients aged 65 and older who presented to the BICU with severe burn injuries. We included data from patients with COPD, T1DM, and T2DM, all of which have increased risk for infections [15-16]. This study aims to identify associations between discharge status (alive/dead) and various patient characteristics with the goal of identifying variables for improved patient care in a rural population.

## Materials and methods

Study design and setting

This retrospective cohort study was conducted at Cabell Huntington Hospital Burn ICU, a tertiary care center serving a predominantly Appalachian population. The study focused on elderly burn patients aged 65 years and older admitted to the facility between January 2017 and January 2023.

Study population

Patients were included if they were aged 65 or older at the time of admission and had sustained burns of any etiology. Patients were excluded if their medical records were incomplete or if they were transferred to another facility before discharge. Data were obtained from the hospital's information technology department, which provided comprehensive information on patients presenting with burns during the study period. Patient data included demographic information (age, gender, and BMI), clinical variables (hospital length of stay, discharge status), comorbid conditions (history of COPD, home oxygen use, smoking history, DM), burn-specific details (source of burn, inhalation injury), and TBSA burned. The TBSA was calculated using the Wallace Rule of Nines. Inhalation injury was diagnosed based on the presence of carbonaceous material or soot in the oropharynx with associated difficulty in oxygenation. Most patients had a bronchoscopy; however, it was not a criterion for diagnosis. Burn sources were categorized into the following types: thermal, chemical and scald. Exclusion criteria included patients misdiagnosed with burns, such as those with Stevens-Johnson syndrome, frostbite, or road rash, as well as cases lacking documented evidence of burns. After exclusion, the final sample consisted of 198 patients aged 65 years and older.

Statistical analysis

Descriptive statistics were used to summarize patient characteristics. Continuous variables were expressed as means and standard deviations or medians and interquartile ranges (IQRs) based on data distribution. Categorical variables were presented as frequencies and percentages. Bivariate analyses were conducted to evaluate associations between discharge status (alive vs. deceased) and independent variables. Chi-square tests were used for categorical variables, such as gender, burn source, and inhalation injury. Welch’s t-test was used for continuous variables with unequal variances, such as age. Mann-Whitney U tests were applied for non-normally distributed variables, such as hospital length of stay and TBS. Statistical significance was set at p < 0.05, with all analyses performed.

## Results

Patient characteristics

Table 1 exhibits an overall simple characteristics of the study population. The study included 198 patients aged ≥65 years, with a mean age of 72.6 ± 6.1 years (range: 65-92). Among the participants, 129 (65.2%) were male, and 69 (34.8%) were female. Most patients survived to discharge (170, 85.9%), while 28 (14.1%) died.

**Table 1:** Patient characteristics This is an overall patient population simple characteristics. %, percentage; SD, standard deviation.

Characteristic	Value
Age (mean ± SD)	72.6 ± 6.1
Age range	65–92
Gender (male)	129 (65.2%)
Gender (female)	69 (34.8%)
Survived	170 (85.9%)
Died	28 (14.1%)

Bivariate analysis

The associations between discharge status (alive vs. death) and each variable are detailed below and exhibited in Table 2: 1) Gender: Among males, 110 (85.3%) survived and 19 (14.7%) died, while among females, 60 (87.0%) survived and nine (13.0%) died. This difference was not statistically significant (p = 0.751, Chi-square test). 2) Age: The mean age of survivors was 72.2 ± 5.9 years, while those who died had a mean age of 74.9 ± 6.4 years. This difference was statistically significant (p = 0.031, Welch’s t-test). 3) Total hospital day: Survivors had a median hospital stay of seven days (IQR: 3-14), while those who died had a median stay of 13 days (IQR: 5-29). The difference was not statistically significant (p = 0.052, Mann-Whitney U test). 4) COPD: COPD was present in 45 (22.7%) patients, of whom 36 (80.0%) survived and nine (20.0%) died. In patients without COPD, 134 (87.0%) survived and 20 (13.0%) died. This difference was not statistically significant (p = 0.179, Chi-square test). 5) Home oxygen use: Among patients using home oxygen (63, 31.8%), 50 (79.4%) survived and 13 (20.6%) died. In patients not using home oxygen (135, 68.2%), 120 (88.9%) survived and 15 (11.1%) died. This difference was not statistically significant (p = 0.257, Chi-square test). 6) Smoking history: Among smokers, 62 (31.3%) patients, 53 (85.5%) survived and 9 (14.5%) died. For non-smokers (136, 68.7%), 117 (86.0%) survived and 19 (14.0%) died. This difference was not statistically significant (p = 0.982, Chi-square test). 7) Diabetes mellitus (DM): Among patients with DM (67, 33.8%), 53 (79.1%) survived and 14 (20.9%) died. In patients without DM (131, 66.2%), 117 (89.3%) survived and 14 (10.7%) died. This difference approached statistical significance (p = 0.083, Chi-square test). 8) Source of burn: The most common source of burn was thermal-fire (101, 51.0%). Mortality rates by burn source were as follows: thermal-fire (13.9%), thermal-explosion (15.8%), scald (0.0%), chemical (33.3%), electrical (0.0%), and other (16.7%). This difference was not statistically significant (p = 0.267, Chi-square test). 9) Inhalation injury: Inhalation injuries were present in 35 (17.7%) patients, of whom 22 (62.9%) survived and 13 (37.1%) died. Among patients without inhalation injuries (163, 82.3%), 148 (90.8%) survived and 15 (9.2%) died. This difference was statistically significant (p < 0.001, Chi-square test). 10) Body mass index (BMI): Survivors had a median BMI of 28.0 (IQR: 24.0-32.0), while those who died had a median BMI of 25.0 (IQR: 23.0-29.0). This difference was not statistically significant (p = 0.073, Mann-Whitney U test). 11) Total burn surface area (TBS): Survivors had a median TBS of 5.0% (IQR: 1.0-10.0), while those who died had a median TBS of 30.0% (IQR: 10.0-50.0). This difference was highly significant (p < 0.001, Mann-Whitney U test). 

**Table 2:** Sample characteristics by survival status. Data presented as n (%), excluding SD. %, percentage; SD, standard deviation; LOS, Length of stay; COPD, chronic obstructive pulmonary disease; DM, diabetes mellitus; BMI, body mass index; TBSA, total body surface area burn. Statistical significance was defined as a p-value of <0.05.

Variable	Survived	Died	p-value
Gender (male)	110 (85.3%)	19 (14.7%)	0.751
Gender (female)	60 (87.0%)	9 (13.0%)	0.751
Age (mean ± SD)	72.2 ± 5.9	74.9 ± 6.4	0.031
LOS (median, IQR)	7 days (3–14)	13 days (5–29)	0.052
COPD	36 (80.0%)	9 (20.0%)	0.179
Home oxygen use	50 (79.4%)	13 (20.6%)	0.257
Smoking history	53 (85.5%)	9 (14.5%)	0.982
DM	53 (79.1%)	14 (20.9%)	0.083
Burn source	87 (86.1%)	14 (13.9%)	0.267
Inhalation injury	22 (62.9%)	13 (37.1%)	<0.001
BMI (median, IQR)	28.0 (24.0–32.0)	25.0 (23.0–29.0)	0.073
TBSA (median, IQR)	5.0 (1.0–10.0)	30.0 (10.0–50.0)	<0.001

## Discussion

Data collected over six years from elderly patients aged 65 and older who were admitted to the only BICU in WV with burn injuries were analyzed. Our study focused on elderly patients in Appalachia due to the aging population and rural territory that makes access to healthcare more challenging [11]. By 2030, the projected number of adults aged 65 and older living in WV will be 25% of the population [11]. The mean age for our study was 72.6 ± 6.1 years, which is higher than the U.S. national burn repository average age of 33 in 2020 [17]. The national mean age is lower as they included pediatric patients in the repository with only 10% of the burn unit admissions being elderly patients [18]. Another study conducted in Morocco found an average age of 76 years in elderly patients admitted to the plastic surgery department in Marrakech [19]. This study had a higher mean age for burn patients but only had a very small sample size of 38 patients [19]. 

Most of the patients in our study were male (n = 129), at 65.2% of the cohort. Discharge status did not vary significantly between males and females in our study (p = 0.751). Other studies found that males have an increased risk for obtaining burn injuries, whereas females are prone to higher mortality rates from severe burn injuries [17,20-21]. This may be due to the inclusion of males age < 18 in other studies who are consistently more prone to burn injuries [21]. The predominance of burn injuries in elderly males may be due to higher occupational hazards working in transportation or industry, which are associated with more severe burns [22]. 

Elderly individuals are more prone to obtaining burn injuries and associated complications due to impaired balance, coordination, and reaction time [7,18]. They usually have a higher TBSA affected, an impaired wound healing process, and thinning of the dermal layer, which leads to deeper burns [8]. These patients are more susceptible to infections and may have cognitive decline, altered mental status, and malnutrition, which leads to worse outcomes [6,9,19]. Infections and pre-existing conditions can play a role in mortality among these patients, but the main predictor of mortality is frailty, which increases with increasing age [6-7,9,19]. In-hospital mortality can be predicted for patients after burn injuries by utilizing the Baux score or revised Baux score [7]. The Baux score formula uses patient age and TBSA to predict mortality, whereas the revised Baux score also includes the presence/absence of inhalation injury and is now used more frequently [9]. Elderly patients tend to have higher Baux scores as age is a factor in the formula, and they have higher rates of mortality in comparison to their younger counterparts after burn injuries [7-8]. Our study found a higher mean age in patients who died, 74.9 ± 6.4 years, in comparison to a mean age of 72.2 ± 5.9 years in survivors (p-value = 0.031). 

The mortality rate in our study was 14.1%, with an age range of 65-92 years. This is lower than the national mortality rate of greater than 25% for fire-related burn injuries in ages > 65 [23]. This does not account for other types of burn injuries such as scald, explosion, electrical, contact, and chemical burns. International studies using cohorts of similar age groups had both higher and lower mortality rates in comparison. A study completed using the Dutch Burn Repository found that the mortality for patients > 65 was 8%, which was much lower than the mortality rate in our study [8]. The higher survival rates in their study may be partially attributed to a shorter median LOS that ranged from 2.4 to 3.5 days in the 65+ age group in comparison to our median LOS in the survival group, 7 days, and the deceased group, 13 days [8]. A Portugal burn unit found a higher mortality rate of 32.3% in patients aged 65-94 who were admitted between 2006 and 2016 [24]. This higher mortality rate can be explained by a higher percentage of patients, 69.2%, with full-thickness burns and a high percentage of these patients, 84.6%, requiring surgery with debridement, skin grafts, flaps, and amputations [24].  

Elderly patients also have more comorbidities involving multiple organ systems upon presentation compared to their younger cohorts and are often taking multiple medications [8,19]. Our study focused solely on patients with comorbidities of diabetes mellitus and COPD. Patients with DM are twice as likely to be hospitalized and to die of infection compared to control groups [15]. They may also have delayed presentation to the hospital after injuries due to diabetic neuropathy and decreased sensation [8,25]. Patients with hyperglycemia are more likely to have concomitant hypertension and respiratory disease [25]. Chronic Obstructive Pulmonary Disease (COPD) is very prevalent in the population. In 2016, COPD was the third leading cause of disability-adjusted life years (DALYs) in the U.S. [26]. Our study found no significant difference in discharge status for patients with COPD (p = 0.179) or DM (p = 0.083) using a Chi-squared test.  

This study also analyzed the discharge status for patients in relation to smoking status (p = 0.982), home oxygen use (p = 0.257), and BMI (p = 0.073). No significant difference was found among any of these variables. Smoking has declined in the U.S. but is an important risk factor to consider for burn injuries [23,26]. Patients with cardiac and pulmonary conditions are often prescribed home oxygen, which is highly flammable and can ignite if not utilized properly [23].  

BMI was also examined due to a greater proportion of individuals affected in rural areas and its increasing prevalence in the U.S. [13,26]. There are limited studies in the literature regarding whether a higher risk for mortality exists for burn patients with increasing BMIs. One study found no association between BMI and hospital mortality rates in elderly patients [27].   

Elderly patients tend to have a longer length of hospital stay compared to younger cohorts after burn injuries [8]. Age and TBSA are main predictors of LOS, but burn depth and other factors may play a role [17]. A prolonged LOS can lead to colonization with antibiotic-resistant strains of microbes, especially in patients in the BICU [28]. Patients with a larger area of skin loss provide a nidus for infection until these areas have healed or until excision and autographing have been performed [1,9]. Infection can also lead to sepsis and death, especially in elderly patients and patients with a higher TBSA due to a dysregulated inflammatory response and hypermetabolic state that occurs post-injury [1-2,9]. This study found no statistical difference in mortality rates for patients that had a prolonged LOS (p = 0.052) but did find a median LOS of 13 days in patients that expired compared to a median LOS of seven days in the survival group. Further studies need to evaluate factors affecting LOS in burn patients and how LOS impacts mortality rates in the elderly [17]. Current research shows that socioeconomic status and median income are also predictors for LOS and recovery in elderly patients [14]. This is essential to provide more consistent patient care and better outcomes. 

This study also analyzed how median TBSA affected discharge status, as an increasing TBSA should lead to worse patient outcomes. This result was found to be significant with a TBS of 30% in patients who expired and a TBSA of 5% in patients who were alive at the time of discharge (p-value < 0.001). Several other studies have found that a higher percentage of TBSA leads to longer hospital stays [3,17]. A higher TBSA also increases the risk for wound infections and complications such as bacterial pneumonia and sepsis, which increases mortality [5]. Elderly individuals with severe burns may also have an impaired wound healing process with uncontrolled inflammation and catabolism, which can lead to organ failure and death [9].  

Mortality rates from different burn sources in our patient demographic were as follows: thermal-fire (13.9%), thermal-explosion (15.8%), scald (0%), chemical (33.3%), electrical (0%), and thermal-contact (16.7%). Thermal-contact and chemical burns had a higher mortality in this population, but these results were not significant (p=0.267). The rural patient demographic in our study tends to have higher rates of poverty, which increases the risk for fire-related burns due to the absence of smoke alarms or their improper maintenance [23]. Elderly individuals living alone also have higher risks for injury [23]. Thermal contact burns in particular, occur from touching hot surfaces such as during cooking and is a leading cause of injuries in the U.S. [23]. Older individuals may be less attentive and acquire unintentional injuries in the home, especially if they have cognitive decline or a decrease in speed when reacting to painful stimuli [23]. These patients may have higher rates of mortality from chemical burns due to prolonged contact with the substance, which results in deeper tissue damage, systemic toxicity, and sepsis [2]. Thermal contact burns with prolonged contact with the hot surface transfer more thermal energy and produce more severe tissue damage [2]. This may increase mortality in these patients.  

In our patient population, inhalation injuries were associated with a higher mortality rate of 37.1% (p < 0.001). The association of higher mortality rates in inhalation injuries are for various reasons, including secondary infections such as pneumonia and acute respiratory distress syndrome (ARDS) [29]. ARDS can develop due to injured mucosa, impaired mucociliary clearance, airway obstruction, and the development of a pro-inflammatory milieu [29]. These patients tend to have higher modified Baux scores due to their age and the fact that they are more likely to have inhalation injuries with slower response times in dangerous scenarios [8]. 

Other studies with an elderly patient population also found higher mortality rates in patients with inhalation injuries. A burn unit in Portugal found a significant difference, p < 0.001, in survival with a mortality rate of 57.1% [24]. The Dutch Burn Repository found a significant increase in inhalation injuries in patients ages 75-82 (5.2%) and in ages 85 and up (6.9%) with a p-value of 0.001% [8]. This study did not examine mortality specifically for inhalation injuries, but these age groups with the presence of more inhalation injuries had higher mortality rates in ages 75-84 (9.1%) and for ages 85 and up (23.8%) with a p-value < 0.001 [8]. 

This retrospective cohort study utilizing data from chart records has some limitations, including incomplete medical records that could not be retrieved from outside facilities, selection bias as the cohort is not representative of elderly patients with burn injuries in the entire population, potential confounding variables, and an inability to infer causation from data obtained. As this was not a nationwide study, the data obtained only represents elderly burn patients seen at Cabell Huntington Hospital in Huntington, WV. This study did not use randomization to minimize confounding variables but did minimize some confounding variables via restriction as participants in the same age group were selected. Bivariate analysis was also used.  

Further research should be conducted to address other variables that may influence hospital discharge status in severe burn patients. It would also be beneficial to conduct a prospective study on outcomes in patients with severe burn injuries in relation to their access to hospital facilities. Until there are more resources allocated to hospitals in rural regions and more facilities equipped to care for burn patients in closer proximity to rural areas, it will remain difficult for patients in these regions to receive adequate, equitable healthcare.  

## Conclusions

This study highlights the significant predictors of survival in elderly burn patients treated in Appalachia, with findings emphasizing the critical roles of age, inhalation injury, and TBSA in determining outcomes. Despite the complex health challenges faced by this population, including high rates of comorbidities and limited healthcare access, these results underscore the importance of focused resource allocation and tailored interventions to improve survival rates. The insights gained from this study can inform clinical practices and policy decisions, particularly in underserved regions, while also providing a foundation for future research aimed at addressing the unique needs of elderly burn patients in rural settings.
